# Available medications used as potential therapeutics for COVID-19: What are the known safety profiles in pregnancy

**DOI:** 10.1371/journal.pone.0251746

**Published:** 2021-05-19

**Authors:** Anick Bérard, Odile Sheehy, Jin-Ping Zhao, Evelyne Vinet, Caroline Quach, Behrouz Kassai, Sasha Bernatsky

**Affiliations:** 1 Research Center, CHU Sainte-Justine, Montreal, Quebec, Canada; 2 Faculty of Pharmacy, University of Montreal, Montreal, Quebec, Canada; 3 Faculty of Medicine, Université Claude Bernard, Lyon, France; 4 Faculty of Medicine, McGill University, Montreal, Quebec, Canada; 5 Faculty of Medicine, University of Montreal, Montreal, Quebec, Canada; University of Insubria, ITALY

## Abstract

**Background:**

Medications already available to treat other conditions are presently being studied in clinical trials as potential treatments for COVID-19. Given that pregnant women are excluded from these trials, we aimed to investigate their safety when used during pregnancy within a unique population source.

**Methods:**

Using the population-based Quebec Pregnancy Cohort, we identified women who delivered a singleton liveborn (1998–2015). Taking potential confounders into account including indications for use, the risk of prematurity, low birth weight (LBW), small for gestational age (SGA), and major congenital malformation (MCM) associated with COVID-19 repurposed drug use during pregnancy were quantified using generalized estimation equations.

**Results:**

Of the 231,075 eligible pregnancies, 107 were exposed to dexamethasone (0.05%), 31 to interferons (0.01%), 1,398 to heparins (0.60%), 24 to angiotensin-receptor blockers (ARB) (0.01%), 182 to chloroquine (0.08%), 103 to hydroxychloroquine (0.05%), 6,206 to azithromycin (2.70%), 230 to oseltamivir (0.10%), and 114 to HIV medications (0.05%). Adjusting for potential confounders, we observed an increased risk of prematurity related to dexamethasone (aOR 1.92, 95%CI 1.11–3.33; 15 exposed cases), anti-thrombotics (aOR 1.58, 95%CI 1.31–1.91; 177 exposed cases), and HIV medications (aOR 2.04, 95%CI 1.01–4.11; 20 exposed cases) use. An increased risk for LBW associated with anti-thrombotics (aOR 1.72, 95%CI 1.41–2.11; 152 exposed cases), and HIV medications (aOR 2.48, 95%CI 1.25–4.90; 21 exposed cases) use were also found. Gestational exposure to anti-thrombotics (aOR 1.20, 95%CI 1.00–1.44; 176 exposed cases), and HIV medications (aOR 2.61, 95%CI 1.51–4.51; 30 exposed cases) were associated with SGA. First-trimester dexamethasone (aOR 1.66, 95%CI 1.02–2.69; 20 exposed cases) and azithromycin (aOR 1.10, 95%CI 1.02–1.19; 747 exposed cases) exposures were associated with MCM.

**Conclusions:**

Many available medications considered as treatments for COVID-19 are associated with adverse pregnancy outcomes. Caution is warranted when considering these medications during the gestational period.

## Introduction

Given the changes to the cardiopulmonary and immune systems during pregnancy, pregnant women are at increased risk for severe COVID-19 [1–3]. In June 2020, the US Centers for Disease Control and Prevention reported that pregnant women with COVID-19 were more likely to be hospitalized and at increased risk for intensive care unit (ICU) admission and receipt of mechanical ventilation compared with non-pregnant women of reproductive age [4]. The World Association of Perinatal Medicine Working Group on COVID-19, with data from Europe, the US, South America, Asia and Australia reported a 0.8% rate of mortality and a 11.1% rate of ICU admissions among infected pregnant women; vertical transmission was negligible [5]. Although vaccination is underway in many countries, pregnant women are often advised against it due to missing data on safety and efficacy during pregnancy. Medications already available to treat other conditions are presently being studied in clinical trials as potential treatments for COVID-19. However, given that pregnant women are excluded from these trials, it is important to assess the current safety profile of these drugs in pregnancy. According to the WHO COVID-19, BMJ COVID-19 Hub, JAMA Network COVID-19, The Lancet COVID-19 Resource Center, New England Journal of Medicine COVID-19, and CMAJ COVID-19 registered trials, these medications include dexamethasone, interferon, heparins, angiotensin-receptor blockers (ARB), chloroquine, hydroxychloroquine, azithromycin, oseltamivir, and HIV medications [6].

At present, results from the COVID-19 RECOVERY trial showed that dexamethasone reduced the mortality rates in severe COVID-19 non-pregnant patients requiring oxygen therapy or on ventilator support [7], as the immunosuppressant dexamethasone may be counteracting the effect of the cytokine in immune dysregulated severe COVID-19 patients [8–11]. Cortisol is critical for embryogenesis, and endogenous fetal glucocorticoid levels remain significantly lower than maternal levels throughout gestation [12,13]; exogenous corticosteroids across the placenta could have adverse developmental effects [14]. Early pregnancy corticosteroid use has been associated with increased risk of orofacial cleft in some [15–20], but not in recent studies [21,22]. Furthermore, studies have reported an increased risk of preterm birth or shorter gestational length following oral corticosteroid use during pregnancy among women with autoimmune disease [23,24].

Likewise, in a phase 2 randomized trial, the immunomodulator interferon beta-1b combined with protease inhibitors (lopinavir–ritonavir) and a nucleoside analogue (ribavirin) was superior to lopinavir–ritonavir alone in reducing the duration of the viral shedding, symptom alleviation, and hospital stay in patients with COVID-19 [25].

About 20%–55% of severe COVID-19 patients have laboratory evidence of coagulopathy [26] and the use of anticoagulant therapy with heparin showed to decrease mortality [27]. Heparin in pregnancy is widely accepted and experienced in women with a high risk of thromboembolism and other conditions; in small sample size studies, heparin use during pregnancy has not shown to be putting the fetus at risk [28].

Angiotensin-converting receptors (ACE2) are required for SARS-CoV2 to enter human cells [29,30]. ACE inhibitors and ARBs are often taken as first-line treatment for hypertension [31], which can result in increased ACE2 expression [32], and increased viral load [33]. Thus, the use of ACE inhibitors or ARBs may aggravate the severity or worsen the outcome of COVID-19 [34,35]. Although ACE inhibitors are contraindicated during pregnancy, ARBs continue to be used inadvertently [36]. At present, ACE inhibitors and ARBs have been shown to be associated with major malformations [36], intrauterine growth retardation, renal dysplasia, anuria, renal failure and death [37–41].

Chloroquine and hydroxychloroquine are currently used to treat and prevent malaria, as well as treat rheumatic diseases. Although many trials have been done on their effectiveness for the treatment of COVID-19, results are conflicting with anecdotal case reports [42–44]. Chloroquine and hydroxychloroquine cross the placenta with a half-life of around 50 days, which could lead to long-term effect during gestation [45]. However, when used for malaria, lupus, or rheumatoid arthritis, hydroxychloroquine was not shown to increase adverse pregnancy outcomes [46]. Other drugs such as azithromycin and oseltamivir, and HIV protease inhibitors indinavir, saquinavir and raltegravir may inhibit the replication of SARS-CoV-2 and have been used in COVID-19 clinical trials [47–50]. Antiretroviral therapy, specifically protease inhibitors, use during pregnancy has been associated with increased risk of preterm birth in some studies [51–59], as antiretroviral therapy produces immunologic changes [60], and interfering with maintenance of pregnancy [61]. A potential safety signal for an increased rate of neural tube defects in association with dolutegravir use in pregnancy has been identified in the surveillance study in Botswana [62], but not in other studies [63–65].

Although immunomodulator dexamethasone and interferon, anticoagulant heparins, angiotensin-receptor blockers (ARB), chloroquine, hydroxychloroquine as well as azithromycin, oseltamivir, and HIV medications are being considered in clinical trials for COVID-19 treatments, their safety in pregnancy need to be determined.

As of now, all these medications have been studied independently in different pregnant populations and not for the treatment of COVID-19 during pregnancy, which makes safety comparisons difficult. Also, these studies would likely not capture pregnant women concomitantly taking more than one COVID-19 potential available treatments, which is highly likely to occur in clinical practice. Finally, different classifications of medication exposure and disease outcomes between studies would lead to imperfect comparisons with regards to safety.

We therefore aimed to quantify the effect of COVID-19 potential available therapeutics, based on the WHO list of registered medication trials, during pregnancy on the risk of prematurity, low birth weight (LBW), small for gestational age (SGA), and major congenital malformations (MCM) using real-world data.

## Methods

### Study cohort

We analyzed data from the Quebec Pregnancy Cohort (QPC), which is a population-based cohort with prospective data collection on all pregnancies covered by the province of Quebec’s universal prescription drug insurance, from 01/01/1998 to 31/12/2015 [66]. Individual-level information for all pregnant women and children are obtained from province-wide databases and linked using unique personal identifiers (S1 Fig). We defined the first day of the last menstrual period (LMP) using data on gestational age, which has been validated against ultrasound measures from each patients’ charts within the QPC [67]. Prospective follow-up is available from 1 year before LMP, during pregnancy, and until 31/12/2015 (S2 Fig).

The QPC data sources include the medical claims database (‘Régie de l’assurance maladie du Québec’ (RAMQ): diagnoses, medical procedures, socio-economic status), Quebec’s outpatient prescription drug insurance database (drug name, start date, dosage, duration), hospitalization archives database (MedEcho: in-hospital diagnoses and procedures, gestational age), and the Quebec birth certificates database (‘Institut de la statistique du Québec’ (ISQ): patient socio-demographics, gestational age, birth weight). Birth weight in ISQ, and MCM and other diagnoses in the RAMQ and MedEcho databases have been found to be valid when compared to patient charts [67,68].

Pregnant women in the QPC were eligible for this study if they were i) more than 18 years old; ii) continuously covered by the Quebec prescription drug insurance for ≥12 months before pregnancy and during pregnancy; and iii) had given birth to a liveborn singleton. This was done because twin pregnancies are at increased risk of adverse pregnancy outcomes regardless of gestational medication exposures. We also excluded pregnancies exposed to known teratogens as described by Kulaga et al. [69] (S1 Table), and those resulting in minor malformations alone or chromosomal abnormalities in the newborns for analyses on MCM. Minor malformations are selectively identified and do not reflect the true prevalence; chromosomal abnormalities are not related to medication use.

#### Ethics statement

The study was approved by the Sainte-Justine’s Hospital Ethics Committee. The Quebec “Commission d’accès à l’information” authorized database linkages. All data were fully anonymized before we accessed them, and the Ethics Committee of CHU Sainte-Justine as well as the ‘Commission d’accès à l’information’ waived the requirement for informed consent.

### Study medication exposures

Study medications included outpatient filled prescriptions of immunomodulator dexamethasone, interferon for multiple sclerosis (beta-1a, beta-1b, and alfa-2b), antithrombotic heparin and heparin derivatives (enoxaparin, dalteparin, and tinzaparin), ARB (losartan and telmisartan), chloroquine, hydroxychloroquine, azithromycin, oseltamivir, and HIV medications (indinavir, lopinavir/ritonavir, saquinavir and raltegravir). We identified study medication prescription fillings from the Quebec prescription drug insurance database (prescribed over-the-counter medications were also included), using timing of exposure determined by the dispensed date and duration of treatment. Pregnancies were dichotomously defined as exposed within each of the study medication groupings if women had filled at least one study medication during pregnancy or if they had filled a prescription with a duration that overlapped the beginning of pregnancy (yes/no). Pregnant women could use more than one study medications during the gestational period, and thus were considered in each corresponding study medication grouping when that was the case. The exposure time window for analyses on prematurity, LBW and SGA was any time during pregnancy; only first trimester exposure (organogenesis) was considered for analyses on MCM.

Data on prescription fillings have been validated and compared to maternal reports in the QPC; the positive predictive value (PPV) of prescription drug data was ≥87% (95%CI: 70%-100%) and the negative predictive value (NPV) was ≥92% (95%CI: 86%-98%) [70].

### Outcomes

Cases of prematurity were identified from the RAMQ and MedEcho databases and defined as deliveries before the 37^th^ week of gestation.

Cases of LBW were identified from the ISQ database as newborns with birthweight less than 2,500g.

Cases of SGA were identified from the MedEcho database (gestational age) and the ISQ database (birth weight and sex) and were defined as birthweights below the 10^th^ percentile for newborns of the same gestational age and the same sex, according to population-based Canadian references [71]. Birth weight in ISQ and gestational age in MedEcho have been found to be valid when compared to patient charts [67,68].

Cases of MCM diagnosed in the first 12 months of life were identified from the RAMQ and MedEcho databases and defined according to ICD-9 and ICD-10 codes (S2 Table), which have been validated against patient charts with high PPV (78.1%) and NPV (94.2%) [68]. All organ systems were considered and high PPV (over 80%) have also been reported for specific MCMs [68], Twelve months after birth was needed to allow for late detection, and validation of early diagnoses.

### Statistical analyses

Within the identified study cohort, we conducted 4 case-control analyses to quantify the effect of the study medication exposures during pregnancy on the occurrence of prematurity, LBW, SGA, and MCM. Although case-control analyses were performed within the study cohort, we have included all controls (no control sampling has been done), and therefore, odds ratios (OR) give the same estimate measure as relative risks.

Potential confounders considered for all analyses were: 1) sociodemographic variables on LMP including maternal age, welfare recipients (yes/no), area of residence (urban/rural); 2) maternal chronic comorbidities (in the 12 months before pregnancy and during pregnancy identified by a diagnosis code or a medication-specific filling) including diabetes, asthma, thyroid disorders (see S3 Table for diagnostic and medication codes used); 3) Tobacco, alcohol, and illicit drug use (See S3 Table); 4) Health care utilization including hospitalizations or emergency department visits during pregnancy (yes/no), number of general practitioner visits and specialist visits (12 months pre-pregnancy); 5) Pregnancy related variables including folic acid use (prescribed high dose (>5 mg/d) and prescribed over-the-counter (OTC) dosage only) in the 6-months prior to LMP and during pregnancy (S3 Table), and previous pregnancy (spontaneous or planned abortion, delivery) in the year prior to LMP (yes/no). We also considered whether pregnant women were followed by an obstetrician (yes/no), and if other medications were used during pregnancy (besides the study medications and medication used to identify comorbidities).

Finally, to control for potential confounding by indication, we adjusted for the presence of the following indications during pregnancy (a pregnant women could have multiple comorbidities): malaria (ICD-9 code 084 and ICD-10 codes B50-B54), lupus (ICD-9 codes 695.4, 710.0 and ICD-10 codes L93, M32), arthritis (ICD-9 codes 274, 696.0, 710.3, 710.4, 714 and ICD-10 codes L40.5, M05, M06, M08, M10, M33.10, M33.20), respiratory tract infections and disorders (ICD-9 codes 011.90, 135, 381, 382, 461, 466, 491.21, 503, 518.3 and ICD-10 codes A15.0, D86, H65-H67, J01-J03, J17, J18, J63.2, J44.1, J82), sexually transmitted diseases and urinary tract infection (ICD-9 codes 077, 099.0, 310, 597–599, 614, 616.0 and ICD-10 codes A31, A54-A57, N34, N37, N70-N77), thrombosis and antiphospholipid syndrome (ICD-9 codes 289.81, 415.19, 444, 453 and ICD-10 codes D68.61, I23, I26, I74, I82), skin disorders (ICD-9 codes 202.1, 694.0, 694.4, 695.1, 695.9 codes and ICD-10 codes C84.0, L10, L13.0, L51.1, L53.9), endocrine disorders (ICD-9 codes 245.0, 255.2, 255.4, 275.4 and ICD-10 codes E06.9, E25.0, E27, E83.52), gastro-intestinal disorders (ICD-9 code 555.9 and ICD-10 codes K50, K51), other hematologic disorders (ICD-9 codes 283, 284, 287.31, 287.4 and ICD-10 codes D59, D60, D61, D69.3, D69.59), human immunodeficiency virus (HIV) (ICD-9 codes 042, 043, 044 and ICD-10 code B20), hepatitis B or C (ICD-9 codes 070.2, 070.3, 070.7 and ICD-10 code B18.2), hypertension (ICD-9 code 401 and ICD-10 code I10), influenza (ICD-9 code 487 and ICD-10 codes J09-J11).

All study medication groupings were always included in analyses, which ensured that estimates were adjusted for concomitant study medication use.

The unit of analysis was a pregnancy. Means and proportions for continuous and dichotomous variables were calculated, respectively. Crude and adjusted odds ratios (aOR) with 95% confidence intervals (95%CI) were calculated for each outcome separately. Multivariable generalized estimating equations were used to estimate the association between the study medications and the risk of prematurity, LBW, SGA, and MCM, independently, accounting for clustering by family (mother). All above mentioned potential confounders and covariables were included in all analyses. All statistical analyses were performed using SAS (SAS Institute Inc., Version 9.2, Cary, NC, USA).

## Results

**Fig 1:**
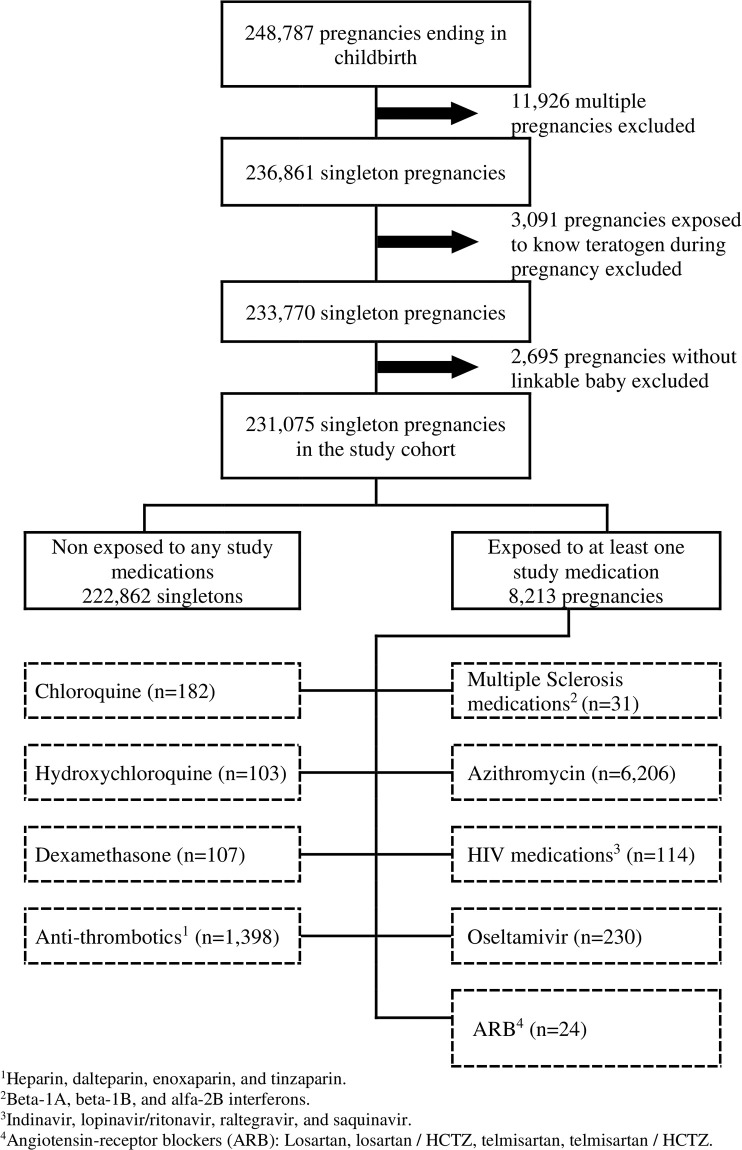
Cohort selection within the Quebec Pregnancy Cohort.

Of the 248,787 pregnancies with a delivery within the QPC, 231,075 met inclusion criteria and were considered for analyses; 8,213 pregnancies were exposed to at least one COVID-19 repurposed drug (Fig 1). We identified 182 pregnancies exposed to chloroquine (0.08%), 103 to hydroxychloroquine (0.05%), 107 to dexamethasone (0.05%), 1,398 to anti-thrombotics (enoxaparin, dalteparin, and tinzaparin, 0.60%), 31 to multiple sclerosis study medications (interferon beta-1a, beta-1b, and alfa-2b, 0.01%), 6,206 to azithromycin (2.70%), 114 to HIV medications (indinavir, lopinavir/ritonavir, raltegravir and saquinavir, 0.05%), 230 to oseltamivir (0.10%), and 24 to the study ARB (losartan and telmisartan, 0.01%) (Fig 1, Table 1).

**Table 1:** Study medication exposures during pregnancy.

		Study medications exposure during pregnancy, n = 8,213								
Characteristics	Non exposed to any of the study drugs	Chloroquine	Hydroxychloroquine	Dexamethasone	Anti-thrombotics^1^	Multiple sclerosis medications^2^	Azithromycin	HIV medications^3^	Oseltamivir	ARB^4^
	n = 222,862	n = 182	n = 103	n = 107	n = 1,398	n = 31	n = 6,206	n = 114	n = 230	n = 24
**At the first day of gestation (1DG)**:										
Mean maternal age (years) ± SD	28.2 ± 5.6	28.8 ± 4.8	31.3 ± 5.6	29.0 ± 6.8	30.1 ± 5.7	28.1 ± 5.2	27.0 ± 6.0	31.0 ± 5.8	29.0 ± 5.2	31.5 ± 5.7
Less than 35	190,320 (85.4)	157 (85.4)	66 (64.1)	83 (77.6)	1,055 (75.5)	29 (93.6)	5,409 (87.2)	82 (71.9)	195 (84.8)	16 (66.7)
35–39	26,761 (12.0)	23 (12.6)	33 (32.0)	17 (15.9)	266 (19.0)	1 (3.2)	644 (10.4)	25 (21.9)	26 (11.3)	7 (29.2)
40 or more	5,781 (2.6)	2 (1.1)	4 (3.9)	7 (6.5)	77 (5.5)	1 (3.2)	153 (2.5)	7 (6.1)	9 (3.9)	1 (4.2)
Welfare recipients—n (%)	50,089 (22.5)	13 (7.1)	33 (32.0)	34 (31.8)	359 (25.7)	12 (38.7)	2,147 (34.6)	59 (51.8)	79 (34.4)	6 (25.0)
Urban dweller—n (%)	183,266 (82.2)	146 (80.2)	87 (84.5)	85 (79.4)	1,135 (81.2)	26 (83.9)	5,301 (85.4)	108 (94.7)	190 (82.6)	18 (75.0)
**Specific indications for the study medication exposures**–n (%)										
Malaria	90 (0.04)	2 (1.10)	0 (0.00)	0 (0.00)	0 (0.00)	0 (0.00)	4 (0.06)	0 (0.00)	1 (0.43)	0 (0.00)
Lupus	118 (0.05)	1 (0.55)	45 (43.69)	2 (1.87)	25 (1.79)	0 (0.00)	8 (0.13)	0 (0.00)	1 (0.43)	0 (0.00)
Arthritis	698 (0.31)	1 (0.55)	52 (50.49)	1 (0.93)	13 (0.93)	0 (0.00)	21 (0.34)	0 (0.00)	2 (0.87)	0 (0.00)
Respiratory track infections	60,349 (27.08)	42 (23.08)	37 (35.92)	35 (32.71)	401 (28.68)	12 (38.71)	2,646 (42.64)	35 (30.70)	75 (32.61)	9 (37.50)
Sexual transmitted disease/UTI^5^	16,338 (7.33)	15 (8.24)	8 (7.77)	13 (12.15)	119 (8.51)	1 (3.23)	639 (10.30)	10 (8.77)	24 (10.43)	4 (16.67)
Behcet’s disease	4 (0.00)	0 (0.00)	0 (0.00)	0 (0.00)	0 (0.00)	0 (0.00)	0 (0.00)	0 (0.00)	0 (0.00)	0 (0.00)
Thrombosis	896 (0.40)	0 (0.00)	2 (1.94)	4 (3.74)	353 (25.25)	1 (3.23)	71 (1.14)	0 (0.00)	4 (1.74)	0 (0.00)
Skin disorders	960 (0.43)	1 (0.55)	7 (6.80)	1 (0.93)	10 (0.72)	0 (0.00)	29 (0.47)	0 (0.00)	2 (0.87)	1 (4.17)
Endocrine disorders	147 (0.07)	0 (0.00)	0 (0.00)	4 (3.74)	5 (0.36)	0 (0.00)	2 (0.03)	2 (1.75)	0 (0.00)	0 (0.00)
Gastrointestinal diseases	1,327 (0.60)	1 (0.55)	2 (1.94)	2 (1.87)	22 (1.57)	0 (0.00)	49 (0.79)	0 (0.00)	1 (0.43)	1 (4.17)
Other hematologic diseases	526 (0.24)	0 (0.00)	1 (0.97)	1 (0.93)	16 (1.14)	0 (0.00)	22 (0.35)	1 (0.88)	1 (0.43)	0 (0.00)
Ankylosing spondylitis	63 (0.03)	0 (0.00)	2 (1.94)	0 (0.00)	0 (0.00)	0 (0.00)	3 (0.05)	0 (0.00)	0 (0.00)	0 (0.00)
Brain tumor	60 (0.03)	0 (0.00)	0 (0.00)	1 (0.93)	2 (0.14)	0 (0.00)	2 (0.03)	0 (0.00)	0 (0.00)	0 (0.00)
HIV^6^	198 (0.09)	2 (1.10)	1 (0.97)	0 (0.00)	0 (0.00)	0 (0.00)	14 (0.23)	94 (82.46)	2 (0.87)	0 (0.00)
Hepatitis	308 (0.14)	1 (0.55)	1 (0.97)	0 (0.00)	3 (0.21)	0 (0.00)	14 (0.23)	1 (0.88)	2 (0.87)	0 (0.00)
Multiple sclerosis	278 (0.12)	0 (0.00)	1 (0.97)	0 (0.00)	8 (0.57)	24 (77.42)	14 (0.23)	0 (0.00)	0 (0.00)	0 (0.00)
Hypertension	5,958 (2.67)	4 (2.20)	5 (4.85)	4 (3.74)	150 (10.73)	0 (0.00)	213 (3.43)	2 (1.75)	6 (2.61)	19 (79.17)
Influenza	6,881 (3.09)	7 (3.85)	3 (2.91)	2 (1.87)	51 (3.65)	2 (6.45)	292 (4.71)	3 (2.63)	72 (31.30)	0 (0.00)
**At delivery**										
Mean duration (weeks) of pregnancy ± SD	38.9 ± 1.8	39.0 ± 2.0	38.2 ± 1.8	38.1 ± 2.7	38.0 ± 2.1	38.8 ± 1.2	38.8 ± 1.8	38.0 ± 2.1	38.8 ± 1.5	38.5 ± 2.0
Mean birthweight (g) ± SD	3352.8 ± 542.3	3387.1 ± 561.4	3102.2 ± 568.6	3188.5 ± 653.6	3158.8 ± 612.5	3232.9 ± 412.2	3306.2 ± 540.0	2985.0 ± 592.7	3316.3 ± 509.7	3235.3 ± 591.5
Prematurity—n (%)	14,310 (6.4)	12 (6.6)	13 (12.6)	15 (14.0)	177 (12.7)	0 (0.0)	472 (7.6)	20 (17.5)	11 (4.8)	2 (8.3)
LBW^7^—n (%)	10,991 (4.9)	9 (5.0)	13 (12.6)	10 (9.4)	152 (10.9)	0 (0.0)	390 (6.3)	21 (18.4)	17 (7.4)	3 (12.5)
SGA^8^—n (%)	21,351 (9.6)	12 (6.6)	16 (15.5)	10 (9.4)	176 (12.6)	3 (9.7)	656 (10.6)	30 (26.3)	26 (11.3)	2 (8.3)
Major congenital malformation—n (%)	22,952 (10.3)	14 (7.7)	16 (15.5)	20 (18.7)	191 (13.7)	2 (6.5)	747 (12.0)	16 (14.0)	29 (12.6)	4 (16.7)
**Maternal comorbidities in the year prior to or during pregnancy**										
Diabetes—n (%)	4,883 (2.2)	4 (2.2)	8 (7.8)	20 (18.7)	107 (7.7)	1 (3.2)	205 (3.3)	7 (6.1)	13 (5.7)	1 (4.2)
Asthma—n (%)	26,099 (11.7)	26 (14.3)	19 (18.5)	21 (19.6)	241 (17.2)	8 (25.8)	1,495 (24.1)	21 (18.4)	43(18.7)	6 (25.0)
Thyroid disorders—n (%)	9,810 (4.4)	9 (5.0)	16 (15.5)	17 (15.9)	96 (6.9)	3 (9.7)	314 (5.1)	3 (2.6)	23 (10.0)	2 (8.3)
Tobacco dependence—n (%)	6,872 (3.1)	2 (1.1)	5 (4.9)	5 (4.7)	71 (5.1)	2 (6.5)	367 (5.9)	2 (1.8)	10 (4.4)	0 (0.0)
Alcohol dependence—n (%)	857 (0.4)	1 (0.6)	1 (1.0)	0 (0.0)	7 (0.5)	0 (0.0)	46 (0.7)	0 (0.0)	2 (0.9)	0 (0.0)
Other drug dependence—n (%)	2,185 (1.0)	1 (0.6)	4 (3.9)	1 (0.9)	21 (1.5)	1 (3.2)	140 (2.3)	9 (7.9)	3 (1.3)	0 (0.0)
**General practitioner visits (in the year prior to pregnancy)**:										
Mean number of visits ± SD	4.4 ± 5.7	4.0 ± 4.6	6.4 ± 7.7	5.6 ± 7.6	5.8 ± 8.2	6.6 ± 6.8	5.8 ± 7.2	5.1 ± 7.6	6.3 ± 8.5	6.3 ± 5.5
Number of visits—n (%)										
0	47,382 (21.3)	30 (16.5)	20 (19.4)	26 (24.3)	266 (19.0)	4 (12.9)	1,094 (17.6)	34 (29.8)	37 (16.1)	2 (8.3)
1	32,809 (14.7)	29 (15.9)	9 (8.7)	13 (12.2)	180 (12.9)	3 (9.7)	738 (11.9)	11 (9.7)	27 (11.7)	1 (4.2)
2–4	69,383 (31.1)	72 (39.6)	23 (22.3)	26 (24.3)	420 (30.0)	6 (19.4)	1,768 (28.5)	28 (24.6)	75 (32.6)	9 (37.5)
≥ 5	73,288 (32.9)	51 (28.0)	51 (49.5)	42 (39.3)	532 (38.1)	18 (58.1)	2,606 (42.0)	41 (36.0)	91 (39.6)	12 (50.0)
**Specialist visits (in the year prior to pregnancy)**:										
Mean number of visists ± SD	3.4 ± 6.2	2.7 ± 3.7	11.4 ± 14.1	7.7 ± 14.4	8.3 ± 11.9	6.7 ± 6.5	4.2 ± 8.6	6.4 ± 7.9	3.7 ± 5.8	2.6 ± 2.6
Number of visits—n (%)										
0	88,122 (39.5)	59 (32.4)	6 (5.8)	27 (25.2)	313 (22.4)	0 (0.0)	2,252 (36.3)	24 (21.1)	80 (34.8)	9 (37.5)
1–2	55,633 (25.0)	59 (32.4)	8 (7.8)	21 (19.6)	255 (18.2)	5 (16.1)	1,437 (23.2)	19 (16.7)	60 (26.1)	4 (16.7)
≥ 3	79,107 (35.5)	64 (35.2)	89 (86.4)	59 (55.1)	830 (59.4)	26 (83.9)	2,517 (40.6)	71 (62.3)	90 (39.1)	11 (45.8)
**Other prescribed medications (during pregnancy)**:										
Mean ± SD	1.7 ± 2.1	2.5 ± 2.5	5.5 ± 3.7	5.5 ± 3.8	4.4 ± 3.8	3.9 ± 3.0	3.4 ± 3.3	5.8 ± 3.4	3.5 ± 3.5	5.4 ± 3.4
Number of medications—n (%)										
0	83,159 (37.3)	28 (15.4)	8 (7.8)	3 (2.8)	83 (5.9)	1 (3.2)	830 (13.4)	0 (0.0)	37 (16.1)	1 (4.2)
1–2	84,932 (38.1)	81 (44.5)	15 (14.6)	20 (18.7)	415 (29.7)	10 (32.3)	2,196 (35.4)	13 (11.4)	76 (33.0)	5 (20.8)
≥ 3	54,771 (24.6)	73 (40.1)	80 (77.7)	84 (78.5)	900 (64.4)	20 (64.5)	3,180 (51.2)	101 (88.6)	117 (50.9)	18 (75.0)
Hospitalization/Emergency Department visit during pregnancy	74,739 (33.5)	53 (29.1)	49 (47.6)	40 (37.4)	690 (49.4)	13 (41.9)	2,697 (43.5)	49 (43.0)	112 (48.7)	9 (37.5)
Prior pregnancy (yes/no)–n (%)	19,236 (8.6)	8 (4.4)	9 (8.7)	10 (9.4)	200 (14.3)	1 (3.2)	580 (9.4)	13 (11.4)	29 (12.6)	0 (0.0)
Current pregnancy follow-up by an obstetrician—n (%)	103,420 (46.4)	84 (46.2)	76 (73.8)	64 (59.8)	1,012 (72.4)	18 (58.1)	2,938 (47.3)	91 (79.8)	134 (58.3)	19 (79.2)
High dose folic acid exposure prior to or during pregnancy—n (%)	8,008 (3.6)	12 (6.6)	24 (23.3)	15 (14.0)	248 (17.7)	6 (19.4)	403 (6.5)	11 (9.7)	27 (11.7)	2 (8.3)

Note: The total number of pregnancies exposed to at least one study medications during pregnancy is equal to 8,213.

^1^Heparin, dalteparin, enoxaparin and tinzaparin.

^2^Beta-1A, beta-1B, and alfa-2B interferons.

^3^Indinavir, lopinavir/ritonavir, raltegravir and saquinavir.

^4^Angiotensin-receptor blockers (ARB): Losartan, losartan/HCTZ, telmisartan, telmisartan/HCTZ.

^5^Urinary tract infection.

^6^Human immunodeficiency viruses.

^7^Low birth weight is defined as birthweight <2500 grams.

^8^ Small for gestational age is defined as birthweight below the 10th percentile for newborns of the same gestational age and same sex.

Study medication users were slightly older; welfare recipients (33.3% vs. 22.5% in non-users); more likely to use high dose (>5 mg/d) folic acid; more likely to have hypertension, diabetes or asthma; and had a higher prevalence of health services utilization including other medication use (Table 1).

Within the study population, 6.5% (15,032) pregnancies resulted in a premature delivery. Adjusting for potential confounders, dexamethasone (aOR 1.92, 95%CI 1.11–3.33; 15 exposed cases), anti-thrombotics (aOR 1.58, 95%CI 1.31–1.91; 177 exposed cases), and HIV medications (aOR 2.04, 95%CI 1.01–4.11; 20 exposed cases) use during pregnancy were statistically significantly associated with an increased risk of prematurity (Table 2A).

**Table 2:** A. Association between study medication exposures during pregnancy and the risk of prematurity. B. Association between study medication exposures during pregnancy and the risk of low birth weight (LBW) (birthweigh <2500 grams). C. Association between study medication exposures during pregnancy and the risk of being born small for gestational age (SGA) (birthweight below the 10th percentile for newborns of the same gestational age and same sex). D. Association between study medication exposures during the first trimester of pregnancy and the risk of overall major congenital malformation.

	Crude OR (95% CI)		Adjusted^1^ OR (95% CI)	
**Study medication exposures (any time during pregnancy)**:				
Chloroquine	1.07	(0.61–1.88)	1.10	(0.62–1.92)
Hydroxychloroquine	2.07	(1.16–3.70)	1.29	(0.67–2.50)
Dexamethasone	2.43	(1.41–4.19)	1.92	(1.11–3.33)
Anti-thrombotics^2^	1.95	(1.64–2.33)	1.58	(1.31–1.91)
Multiple sclerosis medications^3^	N.A.	N.A.	N.A.	N.A.
Azithromycin	1.16	(1.05–1.28)	1.05	(0.95–1.15)
HIV medications^4^	3.05	(1.85–5.01)	2.04	(1.01–4.11)
Oseltamivir	0.71	(0.38–1.31)	0.63	(0.34–1.17)
ARB^5^	1.23	(0.26–5.82)	0.70	(0.15–3.29)
**Indications for study medication exposures (during pregnancy)**:				
Malaria	1.43	(0.70–2.90)	1.35	(0.66–2.75)
Lupus	2.28	(1.50–3.47)	1.67	(1.05–2.64)
Arthritis	1.01	(0.75–1.35)	0.86	(0.65–1.15)
Respiratory track infections	1.01	(0.98–1.05)	0.97	(0.93–1.00)
Sexual transmitted diseases/Urinary tract infections	1.15	(1.08–1.22)	1.07	(1.00–1.14)
Behcet’s disease	N.A.	N.A.	N.A.	N.A.
Thrombosis	1.50	(1.24–1.81)	1.13	(0.92–1.38)
Skin disorders	1.15	(0.91–1.45)	1.10	(0.87–1.40)
Endocrine disorders	1.72	(1.02–2.88)	1.37	(0.80–2.34)
Gastrointestinal diseases	1.49	(1.24–1.79)	1.35	(1.12–1.63)
Other hematologic diseases	1.79	(1.37–2.34)	1.60	(1.23–2.08)
Ankylosing spondylitis	1.21	(0.49–2.96)	1.00	(0.41–2.47)
Brain tumor	1.64	(0.72–3.74)	1.41	(0.63–3.16)
Human immunodeficiency virus	2.08	(1.46–2.96)	1.35	(0.82–2.22)
Hepatitis	1.55	(1.06–2.27)	1.11	(0.75–1.63)
Multiple sclerosis	1.21	(0.80–1.85)	1.04	(0.68–1.57)
Hypertension	2.48	(2.30–2.66)	2.47	(2.25–2.71)
Influenza	1.05	(0.96–1.16)	1.03	(0.94–1.13)
**Sociodemographic variables (at the beginning of pregnancy)**:				
Maternal age (years)				
Less than 35	Ref.		Ref.	
35–39	1.11	(1.06–1.17)	1.12	(1.06–1.17)
40 or more	1.36	(1.25–1.51)	1.33	(1.21–1.46)
Welfare recipients	1.47	(1.42–1.53)	1.37	(1.31–1.42)
Urban dweller	1.00	(0.96–1.05)	0.99	(0.95–1.04)
**Maternal comorbidities in the year prior to the 1 day of gestation or during pregnancy**				
Diabetes	1.81	(1.66–1.99)	1.53	(1.39–1.68)
Asthma	1.20	(1.15–1.26)	1.06	(1.00–1.11)
Thyroid disorders	1.07	(0.99–1.16)	0.98	(0.90–1.06)
Tobacco dependence	1.78	(1.65–1.92)	1.50	(1.38–1.62)
Alcohol dependence	1.95	(1.59–2.40)	1.06	(0.85–1.32)
Other drug dependence	2.44	(2.17–2.75)	1.79	(1.58–2.03)
**Number of general practitioner visits (in the 12 months before pregnancy)**:				
0	Ref.		Ref.	
1	1.02	(0.96–1.08)	1.02	(0.96–1.08)
2–4	1.04	(0.99–1.09)	1.00	(0.95–1.05)
≥ 5	1.24	(1.18–1.29)	1.05	(1.00–1.11)
**Number of specialist visits (in the 12 months before pregnancy)**:				
0	Ref.			Ref.
1–2	1.03	(0.99–1.08)	1.03	(0.99–1.08)
≥ 3	1.27	(1.18–1.29)	1.20	(1.14–1.25)
**Number of other prescribed medications (during pregnancy)**:				
0	Ref.			
1–2	1.03	(0.99–1.07)	0.97	(0.94–1.01)
≥ 3	1.27	(1.22–1.32)	1.03	(0.98–1.08)
**Hospitalization/Emergency Department visit (during pregnancy)**	1.20	(1.16–1.25)	1.02	(0.97–1.06)
**Current pregnancy follow-up by an obstetrician**	0.82	(0.79–0.84)	0.78	(0.75–0.81)
**Prior pregnancy (yes/no)**	1.12	(1.06–1.18)	0.98	(0.92–1.04)
**High dose folic acid exposure prior to or during pregnancy**	1.36	(1.26–1.47)	1.16	(1.07–1.26)
**Study medication exposures (any time during pregnancy)**:				
Chloroquine	1.09	(0.59–2.02)	1.11	(0.59–2.08)
Hydroxychloroquine	2.61	(1.44–4.72)	1.22	(0.57–2.62)
Dexamethasone	1.93	(1.01–3.70)	1.60	(0.82–3.11)
Anti-thrombotics^2^	2.15	(1.78–2.58)	1.72	(1.41–2.11)
Multiple sclerosis medications^3^	N.A.	N.A.	N.A.	N.A.
Azithromycin	1.24	(1.12–1.38)	1.10	(0.98–1.22)
HIV medications^4^	4.25	(2.62–6.87)	2.48	(1.25–4.90)
Oseltamivir	1.55	(0.98–2.47)	1.38	(0.87–2.21)
ARB^5^	2.80	(0.86–9.10)	1.48	(0.46–4.73)
**Indications for study medication exposures (during pregnancy)**:				
Malaria	1.05	(0.56–1.98)	1.09	(0.44–2.70)
Lupus	2.78	(1.95–3.96)	2.36	(1.43–3.90)
Arthritis	1.05	(0.83–1.32)	0.99	(0.74–1.33)
Respiratory track infections	0.95	(0.93–0.98)	0.93	(0.89–0.97)
Sexual transmitted diseases/Urinary track infections	1.03	(0.98–1.09)	1.05	(0.98–1.12)
Thrombosis	1.25	(1.05–1.48)	1.05	(0.84–1.33)
Skin disorders	0.90	(0.73–1.11)	1.03	(0.78–1.36)
Endocrine disorders	1.42	(0.89–2.25)	1.55	(0.88–2.72)
Gastrointestinal diseases	1.23	(1.04–1.45)	1.25	(1.00–1.55)
Other hematologic diseases	1.23	(0.95–1.60)	1.71	(1.29–2.26)
Ankylosing spondylitis	0.93	(0.37–2.34)	1.01	(0.38–2.68)
Brain tumor	0.80	(0.33–1.95)	0.52	(0.14–1.97)
Human immunodeficiency virus	1.98	(1.46–2.69)	1.58	(0.96–2.59)
Hepatitis	2.06	(1.56–2.73)	1.16	(0.76–1.77)
Multiple sclerosis	1.53	(1.12–2.09)	1.34	(0.86–2.10)
Hypertension	1.37	(1.27–1.48)	3.09	(2.79–3.41)
Influenza	1.02	(0.95–1.11)	0.98	(0.88–1.10)
**Sociodemographic variables (at the beginning of pregnancy)**:				
Maternal age (years)				
Less than 35	Ref.		Ref.	
35–39	1.10	(1.04–1.16)	1.11	(1.04–1.17)
40 or more	1.45	(1.31–1.60)	1.39	(1.26–1.54)
Welfare recipients	1.62	(1.55–1.69)	1.48	(1.41–1.54)
Urban dweller	1.01	(0.96–1.06)	0.99	(0.94–1.04)
**Maternal comorbidities in the year prior to the first day of pregnancy or during pregnancy**				
Diabetes	1.27	(1.13–1.42)	1.04	(0.92–1.17)
Asthma	1.35	(1.28–1.43)	1.19	(1.13–1.26)
Thyroid disorders	1.02	(0.93–1.12)	0.96	(0.88–1.06)
Tobacco dependence	2.49	(2.30–2.69)	2.06	(1.90–2.23)
Alcohol dependence	2.64	(2.16–3.23)	1.17	(0.93–1.46)
Other drug dependence	3.00	(2.66–3.39)	1.99	(1.74–2.28)
**Number of general practitioner visits (in the 12 months before pregnancy)**:				
0	Ref.		Ref.	
1	0.97	(0.91–1.04)	0.99	(0.93–1.06)
2–4	1.01	(0.96–1.07)	1.00	(0.94–1.06)
≥ 5	1.20	(1.14–1.26)	1.06	(1.00–1.13)
**Number of specialist visits (in the 12 months before pregnancy)**:				
0	Ref.		Ref.	
1–2	0.99	(0.94–1.04)	0.99	(0.94–1.04)
≥ 3	1.18	(1.13–1.23)	1.11	(1.05–1.17)
**Number of other prescribed medications (during pregnancy)**:				
0	Ref.		Ref.	
1–2	1.05	(1.01–1.10)	0.98	(0.94–1.03)
≥ 3	1.26	(1.20–1.32)	0.99	(0.94–1.04)
**Hospitalization/Emergency Department visit (during pregnancy)**	1.14	(1.10–1.19)	0.96	(0.92–1.01)
**Current pregnancy follow-up by an obstetrician**	0.87	(0.84–0.91)	0.86	(0.82–0.89)
**Prior pregnancy (yes/no)**	1.12	(1.05–1.19)	1.01	(0.95–1.08)
**High dose folic acid exposure prior to or during pregnancy**	1.43	(1.31–1.55)	1.27	(1.16–1.39)
**Study medication exposures (any time during pregnancy)**:				
Chloroquine	0.70	(0.40–1.21)	0.70	(0.40–1.24)
Hydroxychloroquine	1.61	(0.90–2.87)	0.86	(0.42–1.75)
Dexamethasone	1.01	(0.56–1.83)	0.96	(0.52–1.77)
Anti-thrombotics^2^	1.31	(1.11–1.55)	1.20	(1.00–1.44)
Multiple sclerosis medications^3^	0.98	(0.27–3.57)	0.66	(0.19–2.37)
Azithromycin	1.11	(1.02–1.20)	1.04	(0.95–1.12)
HIV medications^4^	3.34	(2.19–5.10)	2.61	(1.51–4.51)
Oseltamivir	1.21	(0.81–1.80)	1.13	(0.76–1.67)
ARB^5^	0.91	(0.23–3.55)	0.84	(0.22–3.21)
**Indications for study medication exposures (during pregnancy)**:				
Malaria	1.05	(0.56–1.98)	1.03	(0.55–1.93)
Lupus	2.78	(1.95–3.96)	2.91	(1.95–4.35)
Arthritis	1.05	(0.83–1.32)	1.04	(0.82–1.32)
Respiratory track infections	0.95	(0.93–0.98)	0.93	(0.90–0.97)
Sexual transmitted disease/Urinary track infections	1.03	(0.98–1.09)	1.02	(0.97–1.07)
Behcet’s disease	3.18	(0.35–28.66)	3.91	(0.46–33.44)
Thrombosis	1.25	(1.05–1.48)	1.16	(0.97–1.39)
Skin disorders	0.90	(0.73–1.11)	0.90	(0.73–1.11)
Endocrine disorders	1.42	(0.89–2.25)	1.45	(0.91–2.31)
Gastrointestinal diseases	1.23	(1.04–1.45)	1.22	(1.03–1.44)
Other hematologic diseases	1.23	(0.95–1.60)	1.19	(0.92–1.55)
Ankylosing spondylitis	0.93	(0.37–2.34)	0.93	(0.39–2.26)
Brain tumor	0.80	(0.33–1.95)	0.80	(0.33–1.95)
Human immunodeficiency virus	1.98	(1.46–2.69)	1.25	(0.83–1.89)
Hepatitis	2.06	(1.56–2.73)	1.69	(1.26–2.26)
Multiple sclerosis	1.53	(1.12–2.09)	1.60	(1.16–2.20)
Hypertension	1.37	(1.27–1.48)	1.52	(1.39–1.66)
Influenza	1.02	(0.95–1.11)	1.01	(0.93–1.09)
**Sociodemographic variables (at the beginning of pregnancy)**:				
Maternal age (years)				
Less than 35	Ref.		Ref.	
35–39	0.95	(0.91–0.99)	0.96	(0.92–1.01)
40 or more	1.06	(0.97–1.15)	1.06	(0.97–1.15)
Welfare recipients	1.36	(1.31–1.40)	1.29	(1.25–1.33)
Urban dweller	1.01	(0.98–1.05)	1.00	(0.96–1.04)
**Maternal comorbidities in the year prior to the first day of gestation or during pregnancy**:				
Diabetes	0.81	(0.73–0.89)	0.80	(0.71–0.88)
Asthma	1.24	(1.19–1.29)	1.19	(1.14–1.24)
Thyroid disorders	0.92	(0.86–0.99)	0.95	(0.88–1.02)
Tobacco dependence	2.03	(1.90–2.16)	1.83	(1.71–1.96)
Alcohol dependence	1.96	(1.65–2.33)	1.15	(0.96–1.38)
Other drug dependence	2.15	(1.93–2.39)	1.64	(1.47–1.84)
**Number of general practitioner visits (in the year before pregnancy)**:				
0	Ref.			Ref.
1	0.97	(0.93–1.02)	1.00	(0.95–1.04)
2–4	0.95	(0.92–0.99)	0.98	(0.94–1.02)
≥ 5	1.01	(0.97–1.05)	1.03	(0.99–1.08)
**Number of specialist visits (in the year before pregnancy)**:				
0	Ref.			Ref.
1–2	0.93	(0.90–0.96)	0.93	(0.90–0.96)
≥ 3	0.89	(0.86–0.92)	0.88	(0.85–0.91)
**Number of other prescribed medications (during pregnancy)**:				
0	Ref.		Ref.	
1–2	1.04	(1.01–1.08)	1.01	(0.98–1.05)
≥ 3	1.09	(1.05–1.13)	0.99	(0.95–1.03)
**Hospitalization/Emergency Department visit (during pregnancy)**	0.96	(0.94–0.99)	0.96	(0.92–0.99)
**Current pregnancy follow-up by an obstetrician**	0.98	(0.95–1.00)	1.02	(0.99–1.05)
**Prior pregnancy (yes/no)**	0.93	(0.89–0.98)	0.96	(0.91–1.01)
**High dose folic acid exposure prior or during of pregnancy**	1.11	(1.03–1.19)	1.11	(1.04–1.20)
**Study medication exposures (in the first trimester of pregnancy)**:				
Chloroquine	0.73	(0.42–1.25)	0.70	(0.41–1.21)
Hydroxychloroquine	1.55	(0.92–2.63)	1.15	(0.65–2.04)
Dexamethasone	2.00	(1.23–3.25)	1.66	(1.02–2.69)
Anti-thrombotics^2^	1.35	(1.15–1.59)	1.02	(0.86–1.21)
Multiple sclerosis medications^3^	0.58	(0.14–2.36)	0.61	(0.14–2.73)
Azithromycin	1.18	(1.10–1.28)	1.10	(1.02–1.19)
HIV medications^4^	1.41	(0.82–2.44)	0.90	(0.47–1.74)
Oseltamivir	1.24	(0.84–1.84)	1.13	(0.77–1.68)
ARB^5^	1.74	(0.62–4.85)	1.25	(0.45–3.53)
**Indications for study medication exposures**:				
Malaria	1.11	(0.59–2.08)	1.04	(0.55–1.94)
Lupus	1.45	(0.98–2.15)	1.19	(0.78–1.81)
Arthritis	1.11	(0.89–1.38)	1.01	(0.81–1.27)
Respiratory track infections	1.00	(0.97–1.03)	0.96	(0.93–0.99)
Sexual transmitted disease/Urinary track infections	1.04	(0.99–1.10)	0.98	(0.94–1.04)
Behcet’s disease	N.A.	N.A.	N.A.	N.A.
Thrombosis	1.58	(1.36–1.84)	1.39	(1.18–1.62)
Skin disorders	1.10	(0.91–1.34)	1.06	(0.88–1.29)
Endocrine disorders	1.29	(0.81–2.06)	1.08	(0.68–1.73)
Gastrointestinal diseases	1.17	(0.99–1.38)	1.09	(0.92–1.28)
Other hematologic diseases	1.27	(1.00–1.63)	1.16	(0.91–1.49)
Ankylosing spondylitis	1.15	(0.54–2.44)	1.07	(0.50–2.31)
Brain tumor	1.18	(0.55–2.53)	1.10	(0.52–2.34)
Human immunodeficiency virus	1.59	(1.16–2.17)	1.38	(0.95–2.00)
Hepatitis	1.29	(0.92–1.79)	1.10	(0.79–1.54)
Multiple sclerosis	0.83	(0.57–1.22)	0.80	(0.54–1.20)
Hypertension	1.47	(1.37–1.58)	1.40	(1.28–1.52)
Influenza	0.92	(0.85–1.00)	0.90	(0.83–0.97)
**Sociodemographic variables (at the beginning of pregnancy)**:				
Maternal age (years)				
Less than 35	Ref.		Ref.	
35–39	1.01	(0.97–1.05)	0.97	(0.93–1.01)
40 or more	1.06	(0.97–1.15)	0.98	(0.91–1.07)
Welfare recipients	1.06	(1.02–1.09)	1.00	(0.97–1.03)
Urban dweller	1.10	(1.06–1.14)	1.08	(1.04–1.12)
**Maternal comorbidities in the year prior to the first day of pregnancy or during pregnancy**				
Diabetes	1.50	(1.39–1.63)	1.28	(1.18–1.38)
Asthma	1.15	(1.11–1.20)	1.08	(1.04–1.13)
Thyroid disorders	1.20	(1.13–1.28)	1.12	(1.05–1.19)
Tobacco dependence	1.26	(1.17–1.35)	1.20	(1.12–1.30)
Alcohol dependence	1.22	(1.00–1.49)	0.95	(0.77–1.16)
Other drug dependence	1.48	(1.32–1.67)	1.34	(1.19–1.51)
**Number of general practitioner visits (in the year prior to the first day of pregnancy)**:				
0	Ref.			
1	1.03	(0.99–1.08)	1.02	(0.98–1.07)
2–4	1.06	(1.02–1.10)	1.03	(0.99–1.07)
≥ 5	1.11	(1.07–1.16)	1.02	(0.98–1.07)
**Number of specialist visits (in the year prior to the first day of pregnancy)**:				
0	Ref.			
1–2	1.05	(1.01–1.08)	0.99	(0.96–1.03)
≥ 3	1.13	(1.10–1.17)	0.97	(0.93–1.01)
**Number of other prescribed medications (during pregnancy)**:				
0	Ref.			
1–2	1.01	(0.97–1.04)	0.99	(0.96–1.02)
≥ 3	1.23	(1.19–1.28)	1.13	(1.09–1.17)
**Hospitalization/Emergency Department visit during pregnancy**	1.18	(1.15–1.22)	1.15	(1.11–1.19)
**Current pregnancy follow-up by an obstetrician**	1.19	(1.16–1.22)	1.18	(1.15–1.22)
**Prior pregnancy (yes/no)**	1.09	(1.04–1.14)	1.02	(0.97–1.07)
**High dose folic acid exposure prior to or during the 1st trimester of pregnancy**	1.34	(1.26–1.42)	1.18	(1.11–1.26)

Note: The total number of pregnancies exposed to at least one study medications during pregnancy is equal to 8,213.

^1^Adjusted for all variables included in the model.

^2^Heparin, dalteparin, enoxaparin and tinzaparin.

^3^Beta-1A, beta-1B, and alfa-2B interferons.

^4^Human immunodeficiency virus medications (Indinavir, lopinavir/ritonavir, raltegravir and saquinavir)

^5^Angiotensin-receptor blockers (ARB): Losartan, losartan/HCTZ, telmisartan, telmisartan/HCTZ.

LBW has been identified in 5.0% (11,606) of newborns. Adjusting for potential confounders including indication for use, anti-thrombotics (aOR 1.72, 95%CI 1.41–2.11; 152 exposed cases), and HIV medications (aOR 2.48, 95%CI 1.25–4.90; 21 exposed cases) were statistically significantly associated with an increased risk of LBW (Table 2B).

Nine percent (9.6%, 22,280) of pregnancies resulted in an SGA newborns. Adjusting for potential confounders including indication for use, anti-thrombotics (aOR 1.20, 95%CI 1.00–1.44; 176 exposed cases), and HIV medication use (aOR 2.61, 95%CI 1.51–4.51; 30 exposed cases) were statistically significantly associated with an increased risk of SGA (Table 2C).

Overall MCM were identified in 10,4% (23,991) of pregnancies. Adjusting for potential confounders, dexamethasone (aOR 1.66, 95%CI 1.02–2.69; 20 exposed cases) and azithromycin (aOR 1.10, 95%CI 1.02–1.19; 747 exposed cases) use during pregnancy were statistically significantly associated with an increased risk of MCM (Table 2D).

Table 3 presents organ specific defects identified with the use of the study medications. Musculoskeletal defects and circulatory malformations including heart defects were the most prevalent in each of the study medication groups. No orofacial defects were found in newborns exposed to dexamethasone in-utero.

**Table 3:** Organ-specific malformations stratified by first-trimester exposures to the study medications.

		Study medication exposures during the first trimester of pregnancy, n = 8,213								
Major congenital malformation by organ system	Non exposed to any of the study drugs	Chloroquine	Hydroxychloroquine	Dexamethasone	Anti-thrombotics^1^	Multiple sclerosis medications^2^	Azithromycin	HIV medications^3^	Oseltamivir	ARB^4^
	n = 222,862	n = 182	n = 103	n = 107	n = 1,398	n = 31	n = 6,206	n = 114	n = 230	n = 24
Nervous system—n (%)	1,292 (0.6)	2 (1.1)	2 (1.9)	1 (0.9)	17 (1.2)	0 (0.0)	43 (0.7)	0 (0.0)	1 (0.4)	0 (0.0)
Eye, ear, face—n (%)	1,023 (0.5)	0 (0.0)	0 (0.0)	0 (0.0)	12 (0.9)	0 (0.0)	29 (0.5)	0 (0.0)	2 (0.9)	0 (0.0)
Circulatory system—n (%)	4,924 (2.2)	3 (1.7)	5 (4.9)	6 (5.6)	55 (3.9)	0 (0.0)	157 (2.5)	4 (3.5)	5 (2.2)	1 (4.2)
Respiratory system—n (%)	1,117 (0.5)	0 (0.0)	0 (0.0)	2 (1.9)	6 (0.4)	0 (0.0)	35 (0.6)	0 (0.0)	1 (0.4)	0 (0.0)
Orofacial, clefts—n (%)	337 (0.2)	1 (0.6)	0 (0.0)	0 (0.0)	0 (0.0)	0 (0.0)	16 (0.3)	0 (0.0)	1 (0.4)	0 (0.0)
Digestive system—n (%)	1,598 (0.7)	0 (0.0)	2 (1.9)	3 (2.8)	17 (1.2)	0 (0.0)	61 (1.0)	1 (0.9)	3 (1.3)	1 (4.2)
Genital system—n (%)	1,707 (0.8)	0 (0.0)	0 (0.0)	0 (0.0)	12 (0.9)	0 (0.0)	51 (0.8)	0 (0.0)	0 (0.0)	1 (4.2)
Urinary system—n (%)	1,878 (0.8)	0 (0.0)	0 (0.0)	1 (0.9)	14 (1.0)	0 (0.0)	65 (1.1)	2 (1.8)	7 (3.0)	0 (0.0)
Musculoskeletal system—n (%)	10,029 (4.5)	7 (3.9)	8 (7.8)	5 (4.7)	68 (4.9)	1 (3.2)	336 (5.4)	9 (7.9)	8 (3.5)	0 (0.0)

^1^Heparin, dalteparin, enoxaparin and tinzaparin.

^2^Beta-1A, beta-1B, and alfa-2B interferons.

^3^Indinavir, lopinavir/ritonavir, raltegravir and saquinavir.

^4^Angiotensin-receptor blockers (ARB): Losartan, losartan/HCTZ, telmisartan, telmisartan/HCTZ.

## Discussion

Using the population-based Quebec Pregnancy Cohort, we quantified the risk of adverse perinatal outcomes associated with available medications presently considered as COVID-19 treatments. Indeed, after adjusting for potential confounders including current indications for use, and concomitant COVID-19 potential therapeutic use, we found that anti-thrombotics, mostly heparins, and HIV medication use during pregnancy were associated with the risk of prematurity, LBW and SGA. Dexamethasone was associated with increasing risks of prematurity and MCM; and azithromycin was associated with the risk of MCM.

This study adjusted for all known and measurable potential confounding variables and estimates are comparable given that they emerge from the same source population, health insurance coverage, and access to care.

Our results on dexamethasone are consistent with the literature with regards to prematurity [23,24]. Palmsten et al. [24] showed that oral corticosteroid use during pregnancy was associated with a doubling of the risk of preterm birth in women with rheumatoid arthritis recruited within teratogen information services (MotherToBaby). Early pregnancy corticosteroid use has also been associated with increased risk of MCM [15–20] similar to what we have shown. We found no orofacial defect with dexamethasone use as was reported in more recent pregnancy studies [21,22].

Our findings on anti-thrombotics (mostly heparins) use and pregnancy are different from what has been published recently. The increased prevalence of adverse fetal/infant outcomes including death, prematurity and MCM have been reported following heparin use [72]. However, in another more recent study performed by Shlomol et al. [28], no such associations were found within an Israeli cohort of pregnant women. While heparin does not appear to cross the placenta, it may affect embryo and fetal development through interactions with the trophoblast and placental vasculature [73]. Differences between our findings and those from Shlomol et al. [28] could be partly explained by their lack of adjustment for potential confounders such as gestational hypertension and diabetes, indications for heparin use, and lifestyles such as tobacco and alcohol use.

Our findings on the association between indinavir, lopinavir/ritonavir, raltegravir and saquinavir (HIV drugs) use during pregnancy and the risk of prematurity, LBW and SGA [51]; and on chloroquine, and hydroxychloroquine with regards to prematurity, LBW or MCM are also consistent with the literature [46].

We found that azithromycin use was increasing the risk of MCM. A recent population-based cohort study using data from the Clinical Practice Research Datalink in the United Kingdom has shown that use of macrolide antibiotics, including erythromycin, clarithromycin, or azithromycin, during pregnancy was associated with an increased risk of overall major congenital malformations in children [74]. Similarly, a population based cohort study using data from the Swedish Medical Birth Register has shown an association between early pregnancy erythromycin use and infant cardiovascular defects [75].

### Strengths and potential limitations

Study strengths include the use of population-based prospective pregnancy cohort with linkage of data at the individual level, which minimized selection and recall biases; this also allowed for analyses on a large number of pregnancies with detailed information regarding exposure, outcomes, and potential confounders. The fact that all potential available medications for COVID-19 treatments were studied within a unique and single population allowed for comparative safety assessments. QPC data on filled prescriptions [70], gestational age [67], birth weight [67], and MCM [67] have all been validated. Adjustment on all known and measurable potential confounders for adverse pregnancy outcomes was made, including maternal comorbidities, indications for medication uses, lifestyles including smoking, alcohol, illicit drug use, and high dose folic acid intake; adjustment was also made on health services utilization, which is considered a proxy for severity of diseases.

One potential limitation is missing information on over-the-counter (OTC) medication use, and use of medications during in-hospital deliveries. Other than for ibuprofen and acetaminophen use and non-prescribed folic acid use, all other medications will be prescribed. It is possible that some women took folic acid OTC. However, this would lead to non-differential misclassification as it is unlikely that those exposed to the study medications would differ in terms of prevalence of folic acid OTC compared to those who were not exposed. Since the databases only include pregnant women insured by the Prescription Drug Insurance program, generalizability of results to those insured by private drug insurance could be affected. However, validation studies have shown that publicly insured pregnant women have similar characteristics and co-morbidities than those who have private medication insurance [76]. We considered filled prescriptions and not actual intake, but Zhao et al. [70] have shown that prescription filling data in the QPC were valid when compared to maternal report. Although health services utilization was adjusted for and considered a proxy for disease severity, residual confounding by severity of disease could remain. Our estimates could be slightly biased upwards since we only considered deliveries in our analyses as is done in the majority of studies on medications and pregnancy. Finally, the MCM prevalence of 10.3% is higher than what is routinely reported (3–5%) [77]. This could be partly explained by the Founders’ effect in the province of Quebec. [78,79]. It can also be partly explained by the fact that we have included all pregnancies between 1998 and 2015, and we have required that all pregnant women and children be insured by the RAMQ public medication insurance program in order to fully measure medication exposures during pregnancy (we only have medication data on those insured by the RAMQ public medication insurance program). This, in addition to the Founders’ effect, could explain the MCM prevalence. Although our baseline prevalence of MCMs is high, it does not differ among our compared groups, and therefore does not invalidate our findings. This, however, could limit the generalizability of our results.

### Conclusions

Using the population-based Quebec Pregnancy Cohort, gestational exposure to dexamethasone was associated with an increased risk of prematurity and MCM; azithromycin exposure was associated with the risk of MCM, and exposure to anti-thrombotics (mostly heparins), and indinavir, lopinavir/ritonavir, raltegravir and saquinavir (HIV drugs) use during pregnancy were associated with increased risks of prematurity, LBW and SGA. Although these available medications are being considered as treatments for COVID-19, caution is warranted in pregnancy.

## Supporting information

S1 Fig Quebec Pregnancy Cohort database linkage. (DOCX)

S2 Fig Quebec Pregnancy Cohort outcomes and babies. (DOCX)

S1 Table List of known fetotoxic prescribed medications excluded. (DOCX)

S2 Table ICD-9 and ICD-10 diagnostic codes of major congenital malformations. (DOCX)

S3 Table List of diagnostic codes (ICD-9 and ICD-10) and medications used for the covariates. (DOCX)
