# Supplementary material for: Available medications used as potential therapeutics for COVID-19: What are the known safety profiles in pregnancy
Source: PLoS One. 2021 May 19;16(5):e0251746. doi: 10.1371/journal.pone.0251746 (PMC8133446; doi:10.1371/journal.pone.0251746)
Supplement: S1 Fig — (DOCX) [file pone.0251746.s001.docx]

**S1 Fig**. Quebec Pregnancy Cohort database linkage.
