# Supplementary figures and images for: Available medications used as potential therapeutics for COVID-19: What are the known safety profiles in pregnancy
Source: PLoS One. 2021 May 19;16(5):e0251746. doi: 10.1371/journal.pone.0251746 (PMC8133446; doi:10.1371/journal.pone.0251746)

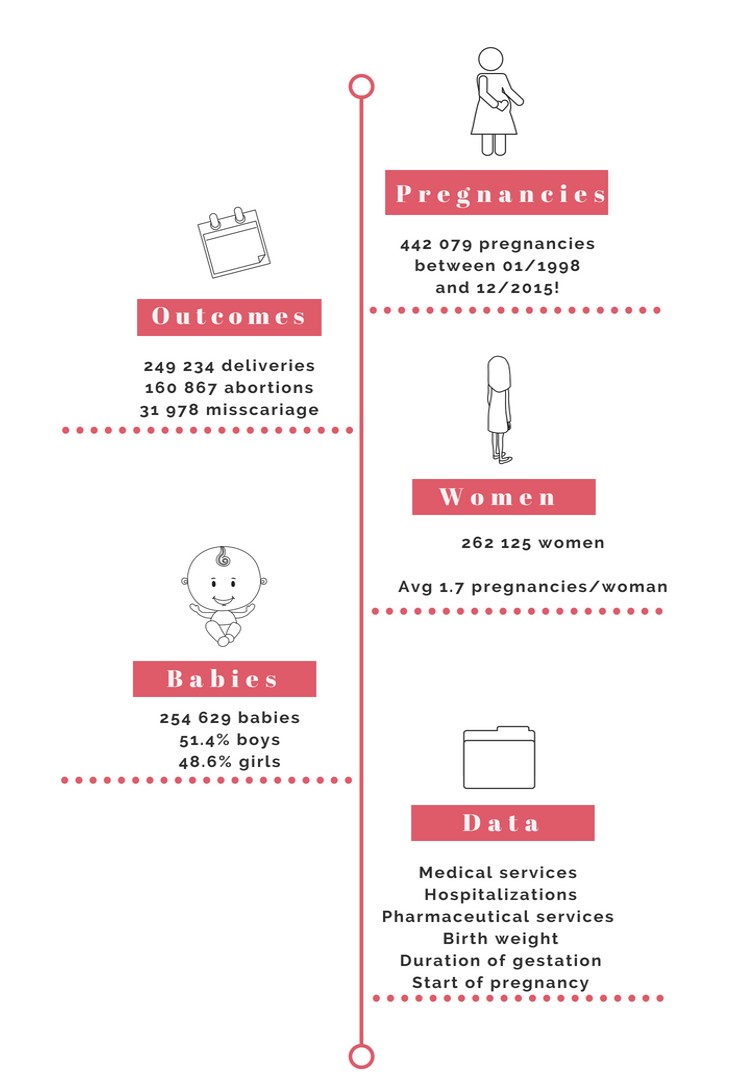


**S2 Fig.** Quebec Pregnancy Cohort outcomes and babies.

Supplement: S2 Fig — (DOCX) [file pone.0251746.s002.docx]
