# Supplementary material for: Available medications used as potential therapeutics for COVID-19: What are the known safety profiles in pregnancy
Source: PLoS One. 2021 May 19;16(5):e0251746. doi: 10.1371/journal.pone.0251746 (PMC8133446; doi:10.1371/journal.pone.0251746)
Supplement: S1 Table — (DOCX) [file pone.0251746.s003.docx]

| **S1 Table. List of known fetotoxic medications excluded** | |
| --- | --- |
| **Class** | **Denomination commune** |
| **systemic retinoids** | isotretinoin, acitretin, etretinate, tretinoin (retinoic acid), retinol, retinal, adapalene, bexarotene, tazarotene |
| **antiepileptics** | Acetazolamide, carbamazepin, eslicarbazepine, ethosuximide, gabapentin, lacosamide, lamotrigine, levetiracetam, oxcarbazepine, perampanel, phenobarbital, phenytoin, pregabalin, primidone, valproic acid, rufinamide, sodium divalproex, sodium valproate, topiramate, sodium valproate, stiripentol, tiagabine, topiramate, vigabatrin, zonisamide |
| **anti-thyroid drugs** | Methimazole, propylthiouracil |
| **anti-coagulants** | Warfarin |
| **tetracycline derivative** | Nicoumalone, doxycyclin, minocyclin, streptomycyn, tetracycline |
| **angiotensin converting enzyme (ACE) inhibitors** | Benazepril, captopril enalapril, fosinopril, lisinopril, perindopril, quinalapril, ramipril, trandolapril, verapamil |
| **androgens** | Danazol, testosterone, methyltestosterone |
| **antineoplastic agents** | Anastrozole, bicalutamide, busereline, busulfan, chlorambucil, cyclophosphamide, estramustine, etoposide, exemestane, fluorouracile, flutamide, gosereline, hydrosyuree, ifosfamide, interferon alfa-2B, interferon alfa-2B (without albumine), letrozole, leuprolide (acetate), melphalan, mercaptopurine, methotrexate, nilutamide, procarbazine, tamoxifen, thioguanine, tretinoin, triptoreline |
| **others** | Amiodarone, diethylstilbestrol, dihydroergotamine, fluconazole, leflunomide, lithium (carbonate), lithium (citrate), methotrexate, penicillamine, cyclophosphamide, misoprostol, diclofenac/misoprostol, quinine |
| **statins** | Atorvastatine calcique, amlodipine/atorvastatine calcique, cerivastatine, fluvastatine sodium, lovastatine, lovastatine/ nicotic acid, pravastatine sodium, pravastatine sodium/AAS, rosuvastatine calcique, simvastatine |
| **angiotensin-receptor blocker (ARB)** | Candersartan, candersartan/HCTZ, eprosartan, eprosartan/HCTZ, irbesartan, irbesartan/HCTZ, olmesartan, olmesartan/HCTZZ, valsartan, valsartan/HCTZ |
