# Supplementary material for: Available medications used as potential therapeutics for COVID-19: What are the known safety profiles in pregnancy
Source: PLoS One. 2021 May 19;16(5):e0251746. doi: 10.1371/journal.pone.0251746 (PMC8133446; doi:10.1371/journal.pone.0251746)
Supplement: S2 Table — (DOCX) [file pone.0251746.s004.docx]

| **S2 Table. ICD-9 and ICD-10 diagnostic codes of major congenital malformations.** | | |
| --- | --- | --- |
| **Congenital malformation** | **ICD-9 diagnostic codes** | **ICD-10 diagnostic codes** |
| Major congenital malformations overall | 740-759 excluding minor congenital malformations  (743.6, 744.1, 744.2-744.4, 744.8, 744.9, 747.0, 747.5, 750.0, 752.4, 752.5, 754.6, 755.0, 755.1, 757.2-757.6, 757.8, 757.9) | Q00-Q89 excluding minor congenital malformations (Q10, Q162, Q17–Q182, Q184–Q189, Q250, Q270, Q381, Q515, Q516, Q520–Q527, Q53, Q664–Q666, Q69, Q70, Q81–Q84, Q950–Q952, Q954, Q955, Q959) |
