# Supplementary material for: Available medications used as potential therapeutics for COVID-19: What are the known safety profiles in pregnancy
Source: PLoS One. 2021 May 19;16(5):e0251746. doi: 10.1371/journal.pone.0251746 (PMC8133446; doi:10.1371/journal.pone.0251746)
Supplement: S3 Table — (DOCX) [file pone.0251746.s005.docx]

**S3 Table. List of diagnostic codes (ICD-9 and ICD-10) and medications used for the covariates**

**Diabetes**

ICD-9 codes: 250.0-250.9, 271.4 and 790.2

ICD-10 codes: E10-E14 and R73.0

Medication generic codes:

| **Generic name** | **Quebec generic code** |
| --- | --- |
| Metformin | 5824 - 47208 |
| Glucagon | 4238 |
| Chlorpropamide | 1937 |
| Glyburide | 4264 |
| Tolbutamide | 9672 - 15184 |
| Gliclazide | 46056 - 47329 |
| Glimepiride | 46799 - 47427 |
| Acarbose | 46300 - 47151 |
| Pioglitazone | 46678 - 47392 |
| Rosiglitazone | 47371 - 46642 |
| Rosiglitazone/Metformine | 46862 |
| Rosiglitazone/Glimepiride | 47652 |
| Nateglinide | 46810 |
| Repaglinide | 47357 - 46568 |
| Saxagliptine | 47817 |
| Sitagliptine | 47715 |
| Sitagliptine/Metformine | 47807 - 47832 |
| Insulin aspart | 46798 - 47424 |
| Insulin aspart/ Insulin aspart protamine |  |
| Insulin glulisine | 47749 |
| Insulin isophane bio-synthetic | 44164 |
| Insulin lispro | 46322 - 47206 |
| Insulin zinc cristalline bio-synthetic | 44489 |
| Insulins zinc cristalline and isophane bio-synthetic | 45531 |
| Insulin aspart/Insulin aspart protamine | 47615 |
| Insulin detemir | 47586 |
| Insulin glargine | 47536 |
| Insulin lispro/Insulin lispro protamine | 47426 |
| Insulin globine zinc | 4823 |
| Insulin sulfate | 4888 |
| Insulin zinc cristalline (porc) | 18296 |
| Insulin protamine zinc (beef) | 18309 |
| Insulin protamine zinc (porc) | 18322 |
| Insulin isophane (porc) | 18335 |
| Insulin isophane (beef) | 18348 |
| Insulin slow release (beef and porc) | 39120 |
| Insulin isophane (beef and porc) | 39133 |
| Insulin protamine zinc (beef and porc) | 39146 |
| Insulin semi-slow release (beef and porc) | 39159 |
| Insulin ultra-slow release (beef and porc) | 39172 |
| Insulin crystal zinc (beef and porc) | 39185 |
| Insulin isophane (beef) | 39458 |
| Insulin protamine zinc (beef) | 39484 |
| Insulin protamine zinc (porc) | 39497 |
| Insulin crystal zinc (beef) | 39523 |
| Insulin slow release (porc) | 41655 |
| Insulin crystal zinc (porc)/ Insulin isophane (porc) | 43033 |
| Insulin crystal zinc (beef) | 43735 |
| Insulin isophane semi-synthetic of human sequence | 44151 |
| Insulin slow release semi-synthetic of human sequence | 44476 |
| Insulin crystal zinc semi-synthetic of human sequence | 44502 |
| Insulin ultra-slow release semi-synthetic of human sequence | 44996 |
| Insulins isophane and crystal zinc semi-synthetics of human sequence | 45405 |
| Insulin slow release bio-synthetic of human sequence | 45415 |
| Insulin ultra-slow release bio-synthetic of human sequence | 45483 |
| Insulins isophane and crystal zinc bio-synthetic of human sequence | 45511 |
| Insulins crystal zinc and isophane semi-synthetics of human sequence | 45534 |
| Insulin crystal zinc (beef and porc) | 46536 |
| Insulin isophane (beef and porc) | 46537 |
| Insulin slow release (beef and porc) | 46538 |
| Insulin isophane(human)/ Insulin injectable (human) | 46592 |
| Insulin isophane (human) | 46602 |
| Insulin injectable (human) | 46603 |
| Insulin lispro/Insulin isophane (human) | 46607 |
| Insulin crystal zinc (porc) | 47004 |
| Insulin lispro/ Insulin lispro protamine | 47426 |
| Alogliptine | 48018 |
| Alogliptine/Metformine | 48017 |
| Canagliflozine | 48013 |
| Dipagliflozine | n.a. |
| Glidazide | n.a. |
| Glimepiride | 47427-46779 |
| Linagliptine | 47881 |
| Linagliptine/Metformine | 47965 |
| Liragludine | n.a. |

**Asthma**

ICD-9 codes: 493.0, 493.1, 493.3, 493.4, 493.5, 493.6, 493.7, 493.8 and 493.9

ICD-10 codes: J45.0, J45.8, J45.1 and J45.9

Medication generic codes

| **Generic name** | **Quebec generic code** |
| --- | --- |
| Aminophylline | 364, 46428 |
| Beclomethasone | 780* |
| Budesonide | 45499* |
| Budesonide/ formoterol | 47428, 46800 |
| Cromoglicate sodium | 39419, 47315 |
| Cromoglycate disodium | 2223 |
| Epinephrine | 3380 |
| Epinephrine | 3406 |
| Epinephrine racemic | 3419 |
| Fenoterol | 38548 |
| Flunisolide | 38730* |
| Fluticasone | 47050*, 46435* |
| Formoterol | 47231* |
| Formoterol | 47271, 46430 |
| Formoterol / budesonide | 47428 |
| Ipratropium (bromure) | 43124, 46640 |
| Ipratropium (bromure)/ salbutamol (sulfate) | 47186, 46302 |
| Isoproterenol (chlorhydrate) | 5083 |
| Isoproterenol (chlorhydrate)/ phenylephrine (bitartrate) | 5096 |
| Isoproterenol (chlorhydrate)/ phenylephrine (chlorhydrate) | 5109 |
| Isoproterenol (sulfate) | 5070 |
| Ketotifene (fumarate) | 45555, 46752 |
| Montelukast sodium | 47303, 47302, 46467 |
| Nedocromil sodium | 47033, 45563, 46463 |
| Orciprenaline (sulfate) | 6721 |
| Oxtriphylline | 43475 |
| Pirbuterol (acetate) | 47153, 46299 |
| Procaterol hemihydrate (chlorhydrate) | 45547 |
| Salbutamol | 10530 |
| Salbutamol (sulfate) | 33634, 46737 |
| Salmeterol (xinafoate) / fluticasone (propionate) | 47335, 46597 |
| Salmeterol (xinafoate) | 47112, 46247 |
| Terbutaline (sulfate) | 34180 |
| Theophylline | 9464, 46847, 9490, 9503 |
| Theophylline/dextrose | 44944 |
| Triamcinolone (acetonide) | 9737* |
| Zafirlukast | 47266, 46401 |
| Ciclesonide | 47626 |
| Mometasone | 45581, 47299 |
| Mometasone/Formeterol | 47884, 47914 |
| Zolair | n.a. |

For code with a* used only the following formulations:

| **Formulation** | **Code** |
| --- | --- |
| Powder aerosol | 1305 |
| Powder aerosol with applicator | 1334 |
| Aerosol solution | 1856 |
| Aerosol solution with applicator | 1885 |
| Solution for Inhalation | 1972 |
| Suspension aerosol | 2610 |
| Suspension aerosol with applicator | 2639 |
| Inhalation powder with applicator | 5563 |
| Inhalation powder | 5564 |
| Oral spray | 5584 |
| Gel | 5619 |
| Powder for solution for inhalation | 5634 |

**Thyroid diseases**

ICD-9 codes: 244.0-244.9 and 242.9

ICD-10 codes: E01, E02, E03 and E05

Medication generic codes:

| **Generic name** | **Quebec generic code** |
| --- | --- |
| Levothyroxine sodium | 5252 - 46574 |
| Liothyronine sodium/ levothyroxine sodium | 33842 |
| Liothyronine sodium | 5317 - 46457 - 46474 |
| Methimazole | 40836 |
| Propylthiouracile | 8242 |

**Tobacco dependence**

ICD-9 codes: 305.0, 305.1 and 649.0

ICD-10 codes: F17, O99.33, Z71.6, and Z72.0

**Alcohol dependence**

ICD-9 codes: 303, 305.0 and 980

ICD-10 codes: F10, O99.31

**Other drugs dependence**

ICD-9 codes: 304.0, 304.2, 304.3, 304.4, 304.5, 304.6, 304.7, 304.8 and 304.9

ICD-10 codes: F11, F12, F14, F15, F16, F18, F19
